# Significance of Endothelial Dysfunction Amelioration for Sodium–Glucose Cotransporter 2 Inhibitor-Induced Improvements in Heart Failure and Chronic Kidney Disease in Diabetic Patients

**DOI:** 10.3390/metabo13060736

**Published:** 2023-06-08

**Authors:** Hidekatsu Yanai, Hiroki Adachi, Mariko Hakoshima, Hisayuki Katsuyama

**Affiliations:** Department of Diabetes, Endocrinology and Metabolism, National Center for Global Health and Medicine Kohnodai Hospital, 1-7-1 Kohnodai, Chiba 272-8516, Japan; dadachidm@hospk.ncgm.go.jp (H.A.); d-hakoshima@hospk.ncgm.go.jp (M.H.); d-katsuyama@hospk.ncgm.go.jp (H.K.)

**Keywords:** endothelial dysfunction, chronic kidney disease, heart failure, sodium–glucose cotransporter 2 inhibitors

## Abstract

Beyond lowering plasma glucose levels, sodium–glucose cotransporter 2 inhibitors (SGLT2is) significantly reduce hospitalization for heart failure (HF) and retard the progression of chronic kidney disease (CKD) in patients with type 2 diabetes. Endothelial dysfunction is not only involved in the development and progression of cardiovascular disease (CVD), but is also associated with the progression of CKD. In patients with type 2 diabetes, hyperglycemia, insulin resistance, hyperinsulinemia and dyslipidemia induce the development of endothelial dysfunction. SGLT2is have been shown to improve endothelial dysfunction, as assessed by flow-mediated vasodilation, in individuals at high risk of CVD. Along with an improvement in endothelial dysfunction, SGLT2is have been shown to improve oxidative stress, inflammation, mitochondrial dysfunction, glucotoxicity, such as the advanced signaling of glycation end products, and nitric oxide bioavailability. The improvements in endothelial dysfunction and such endothelium-derived factors may play an important role in preventing the development of coronary artery disease, coronary microvascular dysfunction and diabetic cardiomyopathy, which cause HF, and play a role in retarding CKD. The suppression of the development of HF and the progression of CKD achieved by SGLT2is might have been largely induced by their capacity to improve vascular endothelial function.

## 1. Introduction

Beyond the lowering of plasma glucose levels, sodium–glucose cotransporter 2 inhibitors (SGLT2is) significantly reduce major adverse cardiovascular events (MACE) in patients with type 2 diabetes and with a history of cardiovascular disease (CVD) or multiple risk factors for CVD [1,2]. The EMPA-REG OUTCOME using empagliflozin, a SGLT2is, showed that empagliflozin significantly reduced three--point MACE, including death from CV causes, nonfatal myocardial infarction, or nonfatal stroke, by 14% compared to the placebo [1]. The EMPA-REG OUTCOME surprised physicians because empagliflozin reduced the hospitalization for heart failure (HF) by 35% when compared with the placebo. A reduction in hospitalization for HF was also observed in the CANVAS Program, which used canagliflozin [2,3]. The DECLARE–TIMI 58 showed that one SGLT2is, dapagliflozin, reduced hospitalization for HF by 27% [4]. Dapagliflozin did not result in a lower rate of MACE than the placebo, but it did result in a lower rate of CV death or hospitalization for HF; there was no between-group difference in CV death. EMPA-REG OUTCOME showed a reduced rate of CV deaths but its effect was driven only by a reduced rate of morbid HF events. The same was shown in the CANVAS Program. There is almost no doubt that SGLT2is can reduce hospitalization for HF, but further investigation is required regarding its effect on MACE.

The difference in the hospitalization for HF between the placebo and SGTL2i appeared from the early phase after SGLT2i administration in all trials. What did these results mean? We think that patients with type 2 diabetes are likely to develop HF, and that SGLT2is improve the factors that exacerbate HF in patients with type 2 diabetes.

The advantage brought by SGLT2is is that they are effective not only in suppressing the onset of HF, but also in suppressing the development and progression of chronic kidney disease (CKD). Empagliflozin reduced incident or worsening nephropathy by 39% compared with the placebo, and decreased the doubling of the serum creatinine level and renal-replacement therapy by 44% and 55%, respectively [5]. Canagliflozin also lowered the renal-specific combination of end-stage renal disease (ESRD), the doubling of the creatinine level, or death from renal causes by 34% [2]. Dapagliflozin reduced the combination of a sustained decline in the estimated glomerular filtration rate (eGFR) by at least 50%, and ESRD or death from renal causes by 44% [6]. SGLT2is such as empagliflozin, canagliflozin and dapagliflozin retarded the decline in eGFR in patients with type 2 diabetes. A short-term (12 weeks) empagliflozin treatment reduced the urinary albumin-to-creatinine ratio (UACR) by 7% in patients with normo-albuminuria, by 25% in patients with microalbuminuria, and by 32% in patients with macroalbuminuria, compared with those who used the placebo. The reductions in UACR were maintained with empagliflozin in all three groups compared with the placebo during a long-term treatment (164 weeks) [7].

Endothelial dysfunction is a very early event in atherosclerosis. Cardiomyocyte is the main player in cardiac function and the development of HF; however, its function is underpinned by non-cardiomyocytes such as vascular endothelial cells. In particular, vascular endothelial cells are important cells for maintaining blood perfusion to myocardial cells. CKD has been found to be an important risk factor not only for ESRD but also for CVD, and the concept of cardiorenal syndrome (CRS) has attracted attention. In CKD patients, systemic vascular endothelial damage is observed from an early stage, which can explain the frequent development of CVD in CKD patients [8].

Endothelial dysfunction is a crucial determinant for the development and progression of ASCVD, HF and CKD, all of which have been improved by SGLT2i use in patients with type 2 diabetes. Here, we discuss the effects of SGLT2is on endothelial dysfunction, and the influence of an improvement in endothelial function owing to SGLT2is on the pathogenesis of ASCVD, HF and CKD.

To understand the association between endothelial dysfunction and diabetes and/or insulin resistance, we searched articles in PubMed, by using the keywords “endothelial dysfunction and diabetes”, “endothelial dysfunction and insulin resistance”, “endothelial function and diabetes”, and “endothelial function and insulin resistance”. To elucidate the etiology that diabetic patients are likely to develop during HF, we searched articles in PubMed by using the keywords “diabetes and heart failure”. As a result of this survey, we found that coronary artery disease (CAD), coronary microvascular dysfunction (CMD) and diabetic cardiomyopathy (DCM) may be the possible mechanisms leading to HF in patients with type 2 diabetes. To understand the association between diabetes and the development of HF, we searched articles in PubMed by using the keywords “endothelial dysfunction and CAD”, “endothelial dysfunction and coronary arterial spasm”, “endothelial dysfunction and CMD”, and “endothelial function and DCM”.

To reveal the association between endothelial dysfunction and CKD, we searched articles in PubMed by using the keywords “endothelial dysfunction and CKD”, “endothelial dysfunction and diabetic kidney disease (DKD)”, and “endothelial dysfunction and diabetic nephropathy”. Furthermore, to understand the effects of SGLT2is on endothelial function, the causative pathological conditions leading to HF, and CKD in patients with type 2 diabetes, we searched articles in PubMed by using the keywords “SGLT2i and endothelial dysfunction”, “SGLT2i and HF”, “SGLT2i and CAD”, “SGLT2i and coronary arterial spasm”, “SGLT2i and CMD”, “SGLT2i and DCM”, “SGLT2i and CKD”, “SGLT2i and DKD”, and “SGLT2i and diabetic nephropathy”. Based on these surveys, we considered the possibility that the improvements in vascular endothelial function produced by SGLT2is are involved in the suppression of the development of HF and CKD progression.

## 2. Endothelial Dysfunction Due to Diabetes and/or Insulin Resistance

**Figure 1:**
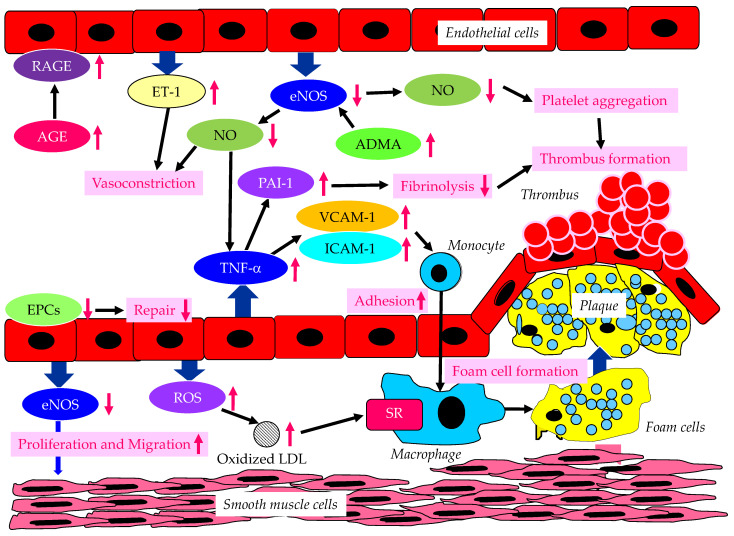
The endothelium-derived molecules and their effects on atherosclerosis, induced by endothelial dysfunction due to diabetes or insulin resistance. AGE, advanced glycation end products; ADMA, asymmetric dimethylarginine; eNOS, endothelial nitric oxide synthase; EPCs, endothelial progenitor cells; ET-1, endothelin-1; ICAM-1, intercellular adhesion molecule-1; LDL, low-density lipoprotein; NO, nitric oxide; PAI-1, plasminogen activator inhibitor-1; RAGE, receptor for advanced glycation end products; ROS, reactive oxygen species; SR, scavenger receptor; TNF-α, tumor necrosis factor-α; VCAM-1, vascular cell adhesion molecule-1. In a diabetic state, AGE and RAGE increase and induce endothelial dysfunction. In a dysfunctional endothelium, a decrease in eNOS and NO results in an increase in TNF-α, which increases VCAM-1, ICAM-1 and PAI-1, which induce vasoconstriction, the adhesion of monocytes to the vascular wall, and thrombus formation. Increased ET-1 also induces vasoconstriction. Decreased eNOS increases ADMA and also induces the proliferation and migration of smooth muscle cells. In dysfunctional endothelial cells, ROS production increases, resulting in an increase in oxidized LDL, which is easily up-taken by macrophages via SR.

The endothelium-derived molecules and their effects on atherosclerosis, induced by endothelial dysfunction due to diabetes or insulin resistance, are shown in Figure 1.

Vascular endothelial dysfunction is an important early stage of atherosclerosis development. Endothelial nitric oxide synthase (eNOS) produces the nitric oxide (NO) in endothelial cells, and eNOS is closely associated with the regulation of anti-atherogenetic processes such as vasorelaxation, an inhibition of the adhesion between leukocytes and endothelial cells, the suppression of the migration and proliferation of vascular smooth muscle cells, and the inhibition of platelet aggregation [9,10,11]. NO promotes vasodilation, and suppresses the proliferation and migration of vascular smooth muscle cells, and suppresses the expression of vascular cell adhesion molecule-1 (VCAM-1) and intercellular adhesion molecule-1 (ICAM-1). Further, NO contributes to the inhibition of cytokine activity, such as tumor necrosis factor-α (TNF-α) and platelet aggregation, and a reduction in procoagulant factors. NO also suppresses the adhesion of monocytes and macrophages to the vascular wall. Elevated TNF-α levels and hyperglycemia are implicated in endothelial dysfunction in patients with diabetes [12,13,14]. TNF-α and hyperglycemia have been reported to elevate plasminogen activator inhibitor-1 (PAI-1) and ICAM-1 and VCAM-1 expression in endothelial cells. PAI-1 and vascular adhesion molecules are elevated in patients with diabetes, which may largely contribute to the pathogenesis of atherosclerosis in diabetic patients [15,16]. Therefore, reduced NO production by endothelial cells induces inflammatory proliferative changes in the vascular wall and allows monocytes to enter the vascular wall, leading to atherosclerotic lesions. In fact, the endothelium-dependent vasorelaxation response is attenuated and vascular endothelial function is impaired due to the decreased activity of eNOS in the vascular walls of patients with insulin resistance [17]. Experiments with endothelial cells have shown that eNOS is activated to produce NO via the insulin-mediated activation of phosphatidylinositol3 (PI3) kinase and the phosphorylation of its downstream Akt [18,19]. Insulin induces NO production by eNOS.

There is growing evidence that the elevated expression of the eNOS inhibitor asymmetric dimethylarginine (ADMA) is associated with the development of endothelial dysfunction [20,21,22]. Further, the elevation of ADMA is associated with an increased risk of CVD. Plasma ADMA levels are positively correlated with insulin resistance in nondiabetic, normotensive people, suggesting a significant association between ADMA and insulin resistance [23].

Endothelial dysfunction is characterized by the enhancement of endothelin-1 (ET-1) expression and the reduced expression of eNOS in endothelial cells. ET-1 is a potent vasoconstrictor, whereas eNOS induces strong vasodilatation via the production of NO [24,25]. Diabetic status induces the formation and accumulation of advanced glycation end products (AGEs). The receptor for AGEs (RAGE) plays a crucial role in the promotion of inflammation and the activation of endothelial cells, which is closely associated with the development and progression of atherosclerosis in patients with diabetes [26,27].

Hyperglycemia may cause the overproduction of mitochondrial reactive oxygen species (ROS), leading to the feed-forward redox stimulation of NADPH oxidases. This vicious cycle may contribute to the development of pathological conditions and facilitate organ damage in diabetes [28]. Such oxidative stress increases the production of oxidized low-density lipoprotein (LDL), which is easily up-taken by macrophages via a scavenger receptor (SR), resulting in foam cell formation.

Endothelial progenitor cells (EPCs) are derived from bone marrow, and can enter blood and differentiate into mature endothelial cells [29]; they play an important role in repairing vascular endothelial damage [30]. Lower EPC levels are significantly associated with a higher CVD incidence in diabetic patients [31].

## 3. A Significance of Endothelial Dysfunction for Development of HF in Patients with Type 2 Diabetes

### 3.1. Patients with Type 2 Diabetes Are Likely to Develop HF?

Diabetes, as well as obesity, is one of the crucial risk factors for HF [32]. The association of glucose metabolism with CV outcome, left ventricular mass (LVM) and LV hypertrophy (LVH) was investigated by using 15,010 subjects with euglycemia, prediabetes and type 2 diabetes in the population-based Gutenberg Health Study [33]. The prevalence of LVH was higher in the order of type 2 diabetes (23.8%), prediabetes (17.8%), and euglycemia (10.2%). The co-prevalence of type 2 diabetes with LVH reduced life expectancy. The development of symptomatic HF, HF hospitalization, and CV death in asymptomatic left ventricular systolic dysfunction patients with and without diabetes was examined [34]. Patients with diabetes had a higher risk of developing HF (hazard ratio [HR], 1.53; 95% confidence interval [95% CI], 1.32 to 1.78; *p* < 0.001), HF hospitalization (HR, 2.04; 95% CI, 1.65 to 2.52; *p* < 0.0001), and the combined outcome of the development of HF or cardiovascular death (HR, 1.48; 95% CI, 1.30–1.69; *p* < 0.001). It was determined whether the risk of adverse CV outcomes associated with diabetes differs in patients with a reduced and preserved ejection fraction of HF (HFrEF and HFpEF) [35]. The prevalence of diabetes was 28.3% in patients with HFpEF and 28.5% in those with HFrEF. Diabetes was associated with a greater relative risk of CV death or HF hospitalization in patients with HFpEF (HR, 2.0; 95% CI, 1.70 to 2.36) than in patients with HFrEF (HR, 1.60; 95% CI, 1.44 to 1.77). In short, diabetes was an independent predictor of CV morbidity and mortality in patients with HF, regardless of EF. Surprisingly, 28% of patients with type 2 diabetes who were not diagnosed with HF had HF, such as HFrEF (5%) and HFpEF (23%) [36]. Such HF-prone characteristics of diabetic patients might have brought an early separation of the curve for HF hospitalization between SGLT2i- and placebo-treated patients in various trials.

### 3.2. The Pathological Conditions Leading to the Development of HF in Patients with Type 2 Diabetes

#### 3.2.1. The Mechanisms Leading to Coronary Artery Disease (CAD) in Patients with Type 2 Diabetes

**Figure 2:**
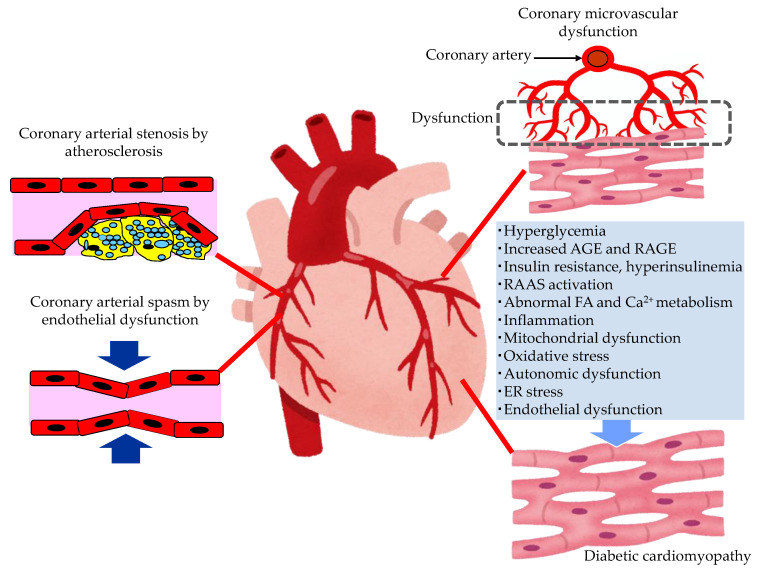
The pathological conditions leading to the development of heart failure in patients with type 2 diabetes. AGE, advanced glycation end products; ER, endoplasmic reticulum; FA, fatty acids; RAAS, renin–angiotensin–aldosterone system; RAGE, receptor for advanced glycation end products.

The pathological conditions leading to the development of HF in patients with type 2 diabetes are shown in Figure 2.

In patients with diabetes, hyperglycemia, insulin resistance, hyperinsulinemia and dyslipidemia, the development of endothelial dysfunction and atherosclerosis is induced. Coronary arterial stenosis, owing to atherosclerosis, causes obstructive CADs, such as angina pectoris. Coronary endothelial dysfunction is thought to be a precursor of obstructive CAD, and is also adversely associated with CV outcomes [37]. In the setting of coronary artery spasm, several clinical studies have demonstrated reduced NO activity, which is observed in endothelial dysfunction [38]. The observation that animal models with mutations of the eNOS gene are predisposed to developing coronary artery spasm further supports the contribution of coronary endothelial dysfunction in the pathogenesis of coronary artery spasm [39].

#### 3.2.2. The Mechanisms Leading Coronary Microvascular Dysfunction (CMD) in Patients with Type 2 Diabetes

Diabetics are often affected by coronary microvascular dysfunction (CMD). This is a condition that consists of a combination of vasomotor changes and long-term structural changes in the coronary arterioles, leading to the dysregulation of blood flow in response to changes in the oxygen demand of myocardial cells [40]. Hyperglycemia, or insulin resistance, may play a central role in leading to oxidative stress, inflammatory activation, and altered endothelial barrier function. CMD contributes significantly to CV events without obstructive CAD, and the development of HF, especially HFpEF, in patients with diabetes.

#### 3.2.3. The Mechanisms Leading to Diabetic Cardiomyopathy (DCM) in Patients with Type 2 Diabetes

Multiple mechanisms, including hyperglycemia, contribute to the development of DCM [41,42,43,44]. In patients with diabetes, the presence of myocardial dysfunction in the absence of overt CAD, valvular disease and other conventional CV risk factors has led to the descriptive terminology, “DCM” [42]. Impaired cardiac insulin resistance, mitochondrial dysfunction, increases in oxidative stress, reduced NO bioavailability, the accumulation of AGEs, impaired mitochondrial and cardiomyocyte calcium handling, inflammation, renin angiotensin–aldosterone system (RAAS) activation, cardiac autonomic dysfunction, and endoplasmic reticulum (ER) stress have all been implicated in the development and progression of DCM. Exposure to increased serum lipid levels, including fatty acids (FA) and triglycerides (TG), causes cardiac lipotoxicity, which is also associated with the development of DCM [43].

Endothelial dysfunction plays a critical role in the onset, development and progression of DCM [45]. Hyperglycemia, hyperinsulinemia, and insulin resistance induce endothelial dysfunction, including the reduced function of the barrier, the impairment of NO bioavailability, the excessive production of ROS, oxidative stress, and inflammation. Endothelial dysfunction induces an impairment of myocardial metabolism, a mishandling of intracellular Ca^2+^, ER stress, mitochondrial dysfunction, the excess production of AGEs, and extracellular matrix deposit. Such various hazardous factors induced by endothelial dysfunction lead to cardiac stiffness, fibrosis, and remodeling, resulting in cardiac diastolic and systolic dysfunction, and the development of HF.

However, we should mention the limitations of our review on the influence of endothelial dysfunction on DCM. Diabetic patients without coronary atherosclerosis and, more importantly, hypertension are few and are usually categorized as low risk according to guidelines. Therefore, DCM as a distinct entity leading to HF is correspondingly rare and, when so, endothelial dysfunction is just one among several other contributing factors that are certainly worth of investigation.

#### 3.2.4. A Significance of Endothelial Dysfunction for Development of Pathogenic Conditions for HF in Patients with Type 2 Diabetes

Endothelial dysfunction plays an important role in the development of CAD, CMD and DCM, which cause HF. Furthermore, HFpEF is a misunderstood disease, for which no mechanisms are clear. Paulus et al. has recently proposed a new hypothesis based on endothelial dysfunction [46]: various comorbidities such as overweight/obesity and diabetes cause endothelial dysfunction, which reduces eNOS functionality and NO production in the endothelial cells. This reduced NO diffuses to the cardiomyocyte less, thus reducing cGMP and hence activating protein kinase G (PKG) less. PKG phosphorylates titin, the main protein responsible for cardiomyocyte stiffness. Titin hypophosphrolylation induced by endothelial dysfunction causes cardiac stiffness and HFpEF. Therefore, endothelial dysfunction causes HFpEF.

## 4. A Significance of Endothelial Dysfunction for Development of CKD in Patients with Type 2 Diabetes

It is known that endothelial dysfunction is not only involved in the onset and progression of CVD, but that it is also an aggravating factor for albuminuria and the progression of renal damage, and the severity of endothelial damage increases with the progression of CKD. Endothelial dysfunction plays a central role in the pathology of CRS. Endothelial dysfunction is deeply involved in renal microvascular hemodynamics, such as the regulation of glomerular filtration and interstitial blood flow, and the maintenance of the vascular network; it also plays an important role in tubulo-glomerular feedback (TGF) and natriuresis. In CKD patients, systemic endothelial dysfunction is observed from an early stage, which may explain why CVD occurs frequently in patients with CKD [7]. The sub-analysis of the Irbesartan in Patients with Type 2 Diabetes and Microalbuminuria (IRMA 2) study showed that endothelial dysfunction was the predicting factor for the progression to diabetic nephropathy in microalbuminuria patients with type 2 diabetes, independent of the traditional risk factors [47]. Flow-mediated vasodilation (FMD) as the marker for endothelial dysfunction was significantly impaired in the patients with elevated urinary albumin excretion compared to normoalbuminuric subjects [48], suggesting a significant association between endothelial dysfunction and albuminuria.

**Figure 3:**
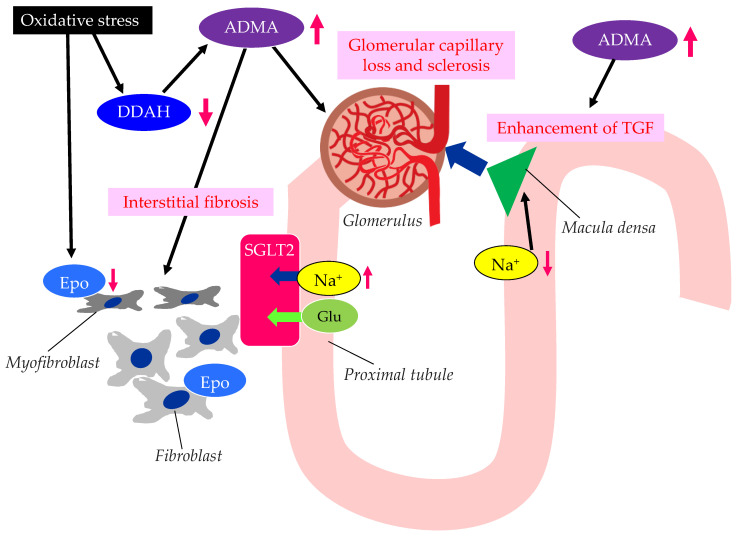
The possible mechanisms leading to CKD in patients with type 2 diabetes. ADMA, asymmetric dimethylarginine; DDAH, dimethylarginine dimethylaminohydrolase; Epo, erythropoietin; Glu, glucose; SGLT2, sodium–glucose cotransporter 2; TGF, tubulo-glomerular feedback. Oxidative stress decreases the function of DDAH. The decreased function of DDAH increases ADMA, which induces renal interstitial fibrosis, glomerular capillary loss and sclerosis, and the enhancement of TGF. The reduced activity of DDAH and an increase in ADMA and oxidative stress induce the formation of dysfunctional fibroblasts, which produce less Epo.

The possible mechanisms leading to CKD in patients with type 2 diabetes are shown in Figure 3.

Elevated levels of oxidative stress and ADMA represent novel risk factors for endothelial dysfunction [49]. There are substantial amounts of data demonstrating that ADMA and oxidative stress markers are elevated in CKD patients [50,51]. Brachial artery endothelium-dependent vasodilatation, which reflects endothelial function, oxidative stress, and ADMA levels, is associated with the stages of CKD [7]. The elevation of plasma and tissue ADMA levels in CKD is induced by both reduced renal excretion and reduced catabolism by dimethylarginine dimethylaminohydrolase (DDAH), which is inhibited by oxidative stress in CKD [52].

ADMA is closely associated with the loss of glomerular capillary and glomerular sclerosis, leading to the progression of CKD [53]. DDAH regulates L-arginine: methylarginine levels in specific renal cells [54] regulate cell-specific L-arginine uptake and NO generation in renal tubular epithelium. The TGF sensitivity is coupled to NO in the macula densa. The TGF was enhanced by ADMA. ADMA has been found to accumulate in the erythrocytes of patients with renal failure [55]. Serum ADMA levels were significantly decreased in CKD patients with anemia and treated with recombinant human erythropoietin (Epo) [56], which may indicate that the activated erythrocyte turnover reduced the accumulation of ADMA in erythrocytes. In such patients, urinary protein levels, the carotid intima–media thickness (IMT), the pulse-wave velocity (PWV), and the plasma brain natriuretic peptide (BNP) level were also significantly decreased. Furthermore, recent studies have shown that erythropoietin protects endothelial function and integrity [57]. Erythropoietin could therefore prevent renal tissue injury and CKD progression.

The ADMA/DDAH may play an important role in the epithelial–mesenchymal transition (EMT) of tubular epithelial cells, which was investigated by using diabetic mice [58]. In the kidneys of diabetic mice, the loss of DDAH induced a higher degree of renal interstitial fibrosis and collagen deposition, and a larger induction of EMT-related changes and oxidative stress than in the kidneys of wild-type mice. Excess oxidative stress induces the injury of the epithelial cells of renal tubules, and injured epithelial cells produce endothelial dysfunction-associated molecules and inflammatory cytokines [59]. The injury of renal tubules induces inflammation via myeloid cells and also induces the transformation of interstitial fibroblasts into myofibroblasts, which leads to renal fibrosis. Such a myofibroblastic transformation induces impaired Epo production by renal interstitial fibroblasts, which causes renal anemia. Such anemia and inflammation induced by the epithelial dysfunction of renal tubules further increase oxidative stress in the kidney, which thus contributes to an unfavorable cycle for the progression of CKD.

Endothelial dysfunction is deeply associated with the development and progression of CKD and diabetic kidney disease (DKD).

## 5. The Effects of SGLT2is on Endothelial Dysfunction

### 5.1. The Effects of SGLT2is on Vascular Function Tests

Noninvasive vascular function tests such as FMD and PWV have been performed to evaluate vascular dysfunction and to identify the individuals at a high risk of CVD [60,61,62]. FMD has been used as a method to assess endothelial function, and PWV has been used as a marker for arterial stiffness.

The addition of dapagliflozin to metformin (16 weeks), when assessed by FMD, improved the endothelial function of patients with poorly controlled early-stage type 2 diabetes [63]. In this study, a reduction in oxidative stress contributed to an improvement in FMD. The two-day treatment with dapagliflozin decreased systolic blood pressure (BP) and oxidative stress [64]. FMD was significantly increased, and PWV was reduced, even after correction for mean BP. Canagliflozin reduced BP and improved arterial stiffness, as assessed by PWV after 6 months, independent of the BP-lowering effect [65]. The effects of SGLT2is on diastolic function and FMD were evaluated in 184 patients with type 2 diabetes and HFpEF [66]. Short-term (12 weeks) SGLT2i treatment improved diastolic function, and with multiple regression, statistically significant associations were seen between the marker for diastolic function and the change in FMD [67]. The 12-month canagliflozin treatment improved diastolic function and FMD in patients with type 2 diabetes and chronic HF (CHF) [67]. The effect of treatment with tofogliflozin for 6 months on cardiac and vascular endothelial function in patients with type 2 diabetes and heart diseases was evaluated. Tofogliflozin treatment (6 months) significantly decreased the left ventricular end-diastolic dimensions and significantly increased FMD [68]. An improvement in diastolic function was significantly correlated with the increase in acetoacetic acid and 3-hydroxybutyrate levels, suggesting that the elevation of ketone bodies by SGLT2is might improve left ventricular dilatation. FMD was significantly improved after the six-month treatment with SGLT2is [69], and multiple regression analysis demonstrated that the change in serum TG was the strongest predictive factor for an improvement in FMD. Switching to SGLT2is was associated with a statistically significant improvement in endothelial function in diabetic patients with CHF after 3 months, and SGLT2i treatment was significantly associated with an improvement in FMD even according to multivariable stepwise regression analysis [70]. A meta-analysis including 26 clinical studies assessing the effects of dipeptidyl peptidase-4 (DPP-4) inhibitors, GLP-1 RAs, and SGLT2is on FMD showed that only SGLT2is significantly improved FMD (mean difference [MD], 1.14%; 95% CI, 0.18 to 1.73, *p* = 0.016), but neither DPP-4 inhibitors (MD, 0.86%; 95% CI: −0.15 to 1.86, *p* = 0.095) nor GLP-1 RA (MD, 2.37%; 95% CI, −0.51 to 5.25, *p* = 0.107) improved FMD [71]. Another meta-analysis including four trials demonstrated that SGLT2is significantly increased FMD by 1.66% (95% CI, 0.56 to 2.76; *p* = 0.003) compared with the placebo or active comparator [72]. Furthermore, SGLT2is improved arterial stiffness in patients with type 2 diabetes [73], and also improved aortic stiffness in nondiabetic patients with HFrEF [74], which is probably explained by the improvement in endothelial function.

However, we should mention that the clinical evidence in this review is weak because the number of studied subjects was very small [63,64]. Similarly, the number of included studies in the meta-analyses was also small [71,72]. Endothelial dysfunction is a generally unspecific phenomenon whose role as a determinant of disease is still unproven. Moreover, basic research data are extrapolated to the clinical situation with difficultly. Further studies should be performed by using a greater number of subjects in order to draw firm conclusions.

We also have to mention the limitations of our review article. We considered PVW and FMD as proxies of the same underlying phenomenon of endothelial dysfunction, but this might not be adequate since PVW is a function of several other and probably more important determinants, such as changes in collagen and elastin, calcification and genetic factors. Endothelial dysfunction is a non-specific abnormality reported in several other clinical conditions (hypertension, obesity, obstructive sleep apnea, sedentary life style unrelated to diabetes) and, in addition, its clinical relevance as a crucial pathogenetic factor has never been demonstrated but only inferred from circumstantial evidence. We should be cautious regarding whether SGLT2is contribute to the amelioration of endothelial function.

### 5.2. The Effects of SGLT2is on Factors-Associated with Endothelial Dysfunction

**Figure 4:**
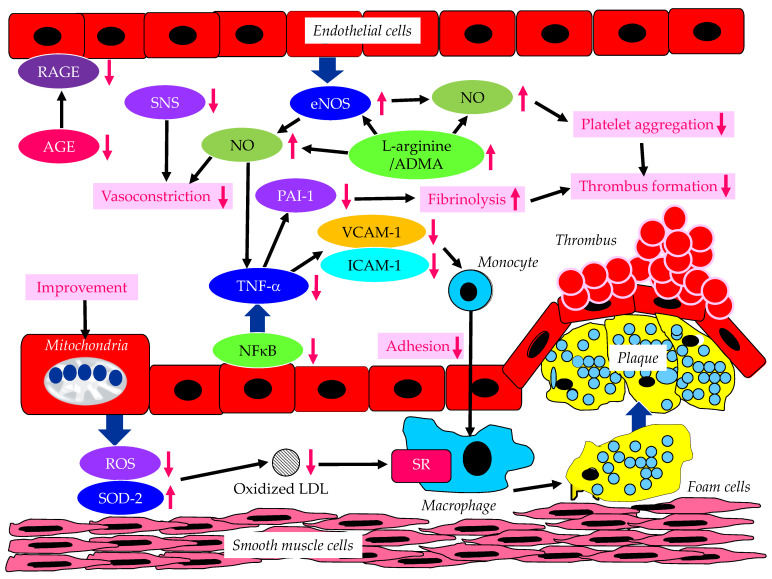
The effects of SGLT2is on endothelium-derived factors induced by endothelial dysfunction. AGE, advanced glycation end products; ADMA, asymmetric dimethylarginine; eNOS, endothelial nitric oxide synthase; ICAM-1, intercellular adhesion molecule-1; LDL, low-density lipoprotein; NFκB, nuclear factor-kappa B; NO, nitric oxide; PAI-1, plasminogen activator inhibitor-1; RAGE, receptor for advanced glycation end products; ROS, reactive oxygen species; SOD-2, super oxide dismutase-2; SR, scavenger receptor; TNF-α, tumor necrosis factor-α; VCAM-1, vascular cell adhesion molecule-1. SGLT2is increase eNOS expression and function, which increase NO and decrease NFκB, TNF-α, PAI-1, VCAM-1 and ICAM-1. SGLT2is increase the L-arginine/ADMA ratio, which also increases eNOS. SGLT2is reduce SNS activity, AGE and RAGE levels. SGLT2is improve mitochondrial function, resulting in a decrease in ROS and an increase in SOD-2, which decreases the formation of oxidized LDL.

The effects of SGLT2is on the factors associated with endothelial dysfunction are shown in Figure 4.

In addition to reducing plasma glucose, empagliflozin normalized endothelial function and ROS in the aorta and blood of diabetic rats [75]. In addition, SGLT2is ameliorates the pro-inflammatory phenotype and glucotoxicity, such as AGE/RAGE signaling, in diabetic animals. Ipragliflozin ameliorated impaired eNOS in the abdominal aorta and reduced ROS generation in diabetic mice [76]. Furthermore, ipragliflozin decreased the expression of VCAM-1 and ICAM-1 in the abdominal aorta. In vitro studies have demonstrated the dapagliflozin-mediated attenuation of TNF-α- and hyperglycemia-induced increases in ICAM-1, VCAM-1, PAI-1 and nuclear factor-kappa B (NFκB) expression [77]. Phlorizin ameliorated the endothelial dysfunction link with the activation of the PI3K/AKT/eNOS signaling pathway and the augmentation of the release of NO, in palmitic acid-induced human umbilical vein endothelial cells [78]. L-arginine is a physiological precursor to the formation of NO. SGLT2i treatment increased the L-arginine/ADMA ratio [79]. The reduced cardiac production of NO and elevated oxidative stress were observed in the ob/ob−/− mice. An increase in the L-arginine/ADMA ratio increased NO bioavailability, improving cardiac contractile function and coronary microvascular function in the ob/ob−/− mice [80]. Empagliflozin and dapagliflozin restored NO bioavailability by inhibiting ROS production rather than affecting eNOS expression/signaling, barrier function, and ICAM-1 and VCAM-1 expression in TNFα-induced endothelial cells [80]. Santos-Gallego, et al. have furthermore demonstrated that empagliflozin improves eNOS functionality, NO production as the ratio of nitrite/nitrate, and all the downstream molecular pathways activated by NO: cGMP–PKG–titin phosphorylation–cardiomyocyte compliance and PKG [81]. This empagliflozin-induced enhancement of NO explains the improvement in endothelial function with SGLT2is.

The epicardial adipose tissue (EAT) contains intrinsic adrenergic and cholinergic nerves, through which it interacts with the cardiac sympathetic and parasympathetic nervous systems [82]. These EAT nerves represent a significant source of bioactive molecules, including norepinephrine, epinephrine, and FA. The abnormal secretion of unfavorable bioactive molecules from EAT is implicated in the development of coronary atherosclerosis and HF. Sympathetic hyperactivity and parasympathetic derangement are associated with EAT dysfunction, thus inducing adverse cardiac conditions, such as HF and diastolic dysfunction [82]. SGLT2is reduced BP, significantly reduced norepinephrine, and improved endothelial function [83], suggesting the beneficial effect of SGLT2i-mediated improvements in the activation of the sympathetic nervous system (SNS) on endothelial function. SGLT2is are considered to induce the suppression of adrenal G protein-coupled receptor kinase-2, which restores/enhances the function of sympatho-inhibitory α2 adrenergic receptors so as to decrease adrenal catecholamine secretion, and downregulates tyrosine hydroxylase to reduce adrenal catecholamine biosynthesis [84]. SGLT2is reduce BP in the absence of increasing heart rate, indicating that SGLT2is may be associated with a reduction in SNS activity. SGLT2 inhibition may lead to a reduction in sympathetic nerve activity, inhibit norepinephrine turnover in brown adipose tissue, and reduce tyrosine hydroxylase production [85].

Empagliflozin reduced frailty in diabetic and hypertensive elderly patients, most likely by decreasing the mitochondrial generation of ROS in endothelial cells [86]. The disruption of the endothelial cell glycocalyx leads to cellular dysfunction, thus promoting inflammation and CVD progression. Empagliflozin mitigated endothelial inflammation and attenuated ER stress signaling caused by sustained glycocalyx disruption [87]. Luseogliflozin ameliorated FA-induced endothelial dysfunction by increasing super oxide dismutase 2 (SOD2) expression and decreasing ROS production in the thoracic aorta of high-fat-diet-induced obese mice [88]. Along with an improvement in kidney function, oxidized LDL, and diastolic function, FMD was significantly increased by canagliflozin in type 2 diabetic patients with CHF [67].

As with various other organ systems/tissues, the important roles that the free FA receptors play in physiology and in various disorders of the cardiovascular system have been revealed [89]. Ketone bodies are a critical cardiac fuel and have diverse roles in the regulation of cellular processes such as metabolism, inflammation, and cellular crosstalk in multiple organs that mediate disease [90]. Growing evidence supports the suggestion that ketone metabolism has an adaptive role in HF and thus helps to promote normal cardiac function and attenuate disease progression [90]. The salutary effects of ketone bodies during HF may also include extra-cardiac roles in modulating immune responses, reducing fibrosis, and promoting angiogenesis and vasodilation. Endothelial dysfunction is also associated with energetic impairment in endothelial cells. SGLT2is cause a metabolic shift in fuel consumption away from energy-inefficient glucose towards the utilization of FA and ketone bodies, which improves energetics. This has been demonstrated in animals [91] and also in humans [92]. Such a mechanism might also explain the improvement in endothelial function with SGLT2is.

## 6. The Effects of SGLT2is on Causative Pathological Conditions Leading to HF in Patients with Type 2 Diabetes

### 6.1. The Effects of SGLT2is on CAD

In a meta-analysis including 22 clinical trials, SGLT2is did not result in any significant differences in the incidence rate of angina pectoris (relative risk [RR], 0.98; 95% CI, 0.83 to 1.14; *p* = 0.92), unstable angina (RR, 0.95; 95% CI, 0.84 to 1.07; *p* = 0.84), or myocardial infarction (RR, 0.94; 95% CI, 0.79 to 1.11; *p* = 0.98) between the SGLT2i and control groups [93]. Another meta-analysis showed that SGLT2is significantly reduce MACE, including hospitalization and all-cause mortality in patients with or without ASCVD, and showed a beneficial trend in patients with HFpEF, and no benefits in patients with stroke or myocardial infarction [94]. A meta-analysis including 15,301 patients with CAD showed that SGLT2is are associated with a reduction in the risk of MACE (HR, 0.84; 95% CI, 0.74 to 0.95), hospitalization for HF (HR, 0.69; 95% CI, 0.58 to 0.83) and a combination of CV death or hospitalization for HF (HR, 0.78; 95% CI, 0.71 to 0.86) in CAD patients [95]. Although no data have so far shown that SGLT2is suppress the onset of CAD, it has been demonstrated that SGLT2is suppress hospitalization for HF in CAD patients.

### 6.2. The Effects of SGLT2is on CMD

SGLT2i treatment ameliorated both cardiac contractile function and coronary microvascular function, as assessed by fractional area change (FAC) and coronary flow velocity reserve (CFVR), respectively, in prediabetic ob/ob−/− mice [79]. Coronary flow reserve (CFR) is regulated not only by focal stenosis, but also by diffuse atherosclerosis and CMD in patients with CAD. The CFR was reduced in the db/db group; however, empagliflozin significantly increased CFR [96]. The number and microvascular coverage of cardiac pericytes were reduced in the db/db mice, but they were improved by empagliflozin. In short, empagliflozin improved CMD and reduced the loss of cardiac pericytes in diabetic mice.

SGLT2is reduced cardiac ischemia-reperfusion injury, and also reduced infarct size and microvascular obstruction [97]. These benefits can be explained by the improvement in endothelial function, especially considering the reduced area of microvascular obstruction in the empagliflozin group. In fact, the microvascular function in the myocardium is worsened by inflammation and improved by empagliflozin [98].

### 6.3. The Beneficial Effects of SGLT2is on DCM

The beneficial effects of SGLT2is on DCM are shown in Table 1. SGLT2is have multiple beneficial factors that improve DCM [99,100,101,102,103,104,105,106,107,108,109,110]. SGLT2is decreased fibrosis, reduced inflammation and improved systolic function. SGLT2is improved diastolic function and reduced mortality in a model of DCM [102]. SGLT2is may be a promising therapeutic option for DCM.

### 6.4. The Effects of SGLT2is on the Development of HF and Mortality

The EMPEROR-Reduced Trial showed that among patients receiving recommended therapy for HF, patients in the empagliflozin group had a lower risk of CV death or hospitalization for HF than those in the placebo group, regardless of the presence or absence of diabetes [111]. Empagliflozin improved nondiabetic HF patients’ quality of life [112]. Empagliflozin significantly improved nondiabetic HF patients’ LV volumes, LV mass, LV systolic function, functional capacity, and quality of life compared with the placebo [113].

A great number of studies have been published on the impact of SGLT2is on hospitalization for HF and mortality. Here, we present a summary of high-evidence-leveled studies, such as meta-analyses and big-database studies, on the impact of SGLT2is on hospitalization for HF and mortality (Table 2).

## 7. The Effects of SGLT2is on CKD

### 7.1. The Underlying Mechanisms for SGLT2i-Induced Improvement of CKD

There are several renal protective mechanisms caused by SGLT2is [125,126,127]. An improvement in metabolic factors, including a reduction in body weight and BP, an increase in insulin sensitivity, and a decrease in serum uric acid, may be associated with SGLT2i-mediated renal protection [128]. Ketone bodies are also used by the diabetic failing myocardium as a super fuel to improve heart function, and an improvement in CRS leads to further improvements in renal function. SGLT2is decrease the overload of the proximal tubules and improve the tubulointerstitial hypoxic milieu, leading to the recovery of Epo production by fibroblasts [129]. Therefore, the increase in hematocrit values caused by SGLT2is suggests the recovery of tubulointerstitial function in DKD [129]. Elevated Epo may also contribute to the renal protective effect of SGLT2is [130]. Treatment with human erythropoietin protected the kidney of streptozotocin-induced diabetic rats [131]. Epo was reported to protect podocytes from injury caused by AGEs in mice [132]. Epo ameliorated the injury of podocytes in advanced DKD in db/db mice [133]. An elevated expression of SGLT2 increased the renal NaCl reabsorption in the proximal tubule, inducing a significant reduction in distal NaCl delivery to the macula densa [134]. The decreased NaCl delivery to the macula densa is sensed as a decrease in plasma volume, which leads to maladaptive glomerular afferent arterial vasodilatation; such abnormally enhanced TGF increases intraglomerular pressure [135] and a worsening of the renal function. SGLT2is restore normal TGF [136], which may reduce albuminuria and maintain eGFR. The above-mentioned renal protective mechanisms caused by SGLT2is are significantly associated with an improvement in endothelial dysfunction.

### 7.2. The Effect of SGLT2is on Renal Outcomes in Patients with Type 2 Diabetes and CKD

The effect of SGLT2is on the renal outcomes of patients with type 2 diabetes and/or CKD is shown in Table 3.

Empagliflozin, dapagliflozin and canagliflozin have been shown to improve renal outcomes.

Several meta-analyses have reported the beneficial effects of SGLT2is on CKD. SGLT2is have been significantly associated with a reduction in albuminuria in patients with type 2 diabetes and CKD [125]. SGLT2is improved the risk of CVD and renal outcomes in patients with type 2 diabetes and CKD [140]. In patients with cardiometabolic and kidney disease, SGLT2is improved CV and kidney outcomes, regardless of type 2 diabetes, HF, and/or CKD status [141]. The magnitude of risk reduction was largest for hospitalization for HF and the progression of CKD. In a meta-analysis that investigated CVD and renal outcomes using SGLT2is vs. GLP-1RAs in type 2 diabetic patients with CKD, SGLT2is were associated with a decreased risk of CVD and renal events, but GLP-1RAs were not [142]. SGLT2is significantly reduced the risk of renal events compared with GLP-1RAs. SGLT2is reduced the risk of renal outcomes and MACE for patients with type 2 diabetes and CKD stage 3b-4 [143]. SGLT2is significantly reduced the risk of primary outcomes that involved the worsening of kidney function, ESRD, or renal death in CKD patients [144].

## 8. Safety Profile of SGLT2is

SGLT2is can cause hypoglycemia, hypotension, volume depletion, lower-limb amputation, fractures, genital infection, urinary infections, and diabetic ketoacidosis with different frequencies of onset [145]. Several adverse events have been reported, whose incidence and severity might be increased in the elderly population [146]. The benefit/risk ratio of SGLT2is in older patients with type 2 diabetes was investigated. Consistent results showed a similar relative risk reduction in CV mortality and HF with SGLT2is, independent of age. The safety profile of SGLT2is appeared comparable in older versus younger patients [146]. Caution may be required in very old frail patients, especially those exposed to an increased risk of volume depletion.

## 9. Conclusions

SGLT2is improve endothelial dysfunction measured by FMD in individuals at a high risk of CVD. The improvements in endothelial dysfunction and endothelium-derived factors may play an important role in preventing the development of CAD, CMD and DCM, which cause HF, and in retarding CKD. The suppression of HF and CKD progression achieved by SGLT2is may be largely due to the improvement of endothelial function owing to SGLT2is.

## Figures and Tables

**Table 1:** The beneficial effects of SGLT2is on DCM.

Beneficial Factors of SGLT2is with Ability to Improve DCM
1. Reduction in endoplasmic reticulum stress 2. Inhibition of oxidative stress 3. Attenuated myocardial fibrosis and apoptosis 4. Reduced inflammation 5. Improvement in myocardial fatty acid and glucose metabolism 6. Improvement in mitochondrial function 7. Attenuated arrhythmogenesis 8. Normalizing intracellular Ca^2+^ handling in cardiomyocytes 9. Inhibition of excessive autophagy 10. Improvement in myocardial energetics

**Table 2:** The meta-analyses and big-database studies on the impact of SGLT2is on hospitalization for HF and mortality.

SGLT2is	Included Studies	Patients	HHF and ACM	HHF	ACM	CVM	Ref.
Empagliflozin	Databases, propensity score-matched analyses comparing with DPP4i	Patients with type 2 diabetes	0.76 (0.67–0.86) * vs. DPP4i	0.82 (0.71–0.94) vs. DPP4i	NA	NA	[114]
	Databases, propensity score-matched analyses comparing with DPP4i	Patients with type 2 diabetes	NA	0.70 (0.60–0.83) vs. DPP4i	0.55 (0.48–0.63) vs. DPP4i	0.59 (0.42–0.84) vs. DPP4i	[115]
	Databases for all related RCTs	Patients with CHF	NA	0.76 (0.69–0.84) vs. placebo	0.96 (0.86–1.08) vs. placebo	0.90 (0.78–1.03) vs. placebo	[116]
	Cardiovascular outcome trials	Patients with type 2 diabetes	NA	0.68 (0.59–0.78) vs. placebo	NA	NA	[117]
	Databases, propensity score-matched analyses comparing with DPP4i	Patients with type 2 diabetes	NA	0.82 (0.71–0.94)	0.64 (0.50–0.81)	NA	[118]
	Databases, propensity score-matched analyses comparing with DPP4i	Patients with type 2 diabetes	NA	0.65 (0.47–0.90)	0.67 (0.54–0.83)	0.60 (0.46–0.79)	[119]
Dapagliflozin	Databases for all related RCTs	Patients with CHF	NA	0.68 (0.58–0.80) vs. placebo	0.77 (0.66–0.91) vs. placebo	0.78 (0.65–0.92) vs. placebo	[116]
	Cardiovascular outcome trials	Patients with type 2 diabetes	NA	0.70 (0.62–0.79) vs. placebo	NA	NA	[117]
	Pooled meta-analysis of two trials including DAPA-HF and DELIVER	Patients with HF	NA	0.71 (0.65–0.78) vs. placebo	0.90 (0.82–0.99) vs. placebo	0.86 (0.76–0.97) vs. placebo	[120]
	RCTs	Patients with type 2 diabetes and HF	NA	0.74 (0.61–0.88) vs. placebo	0.76 (0.66–0.94) vs. placebo	0.84 (0.69–1.03) vs. placebo	[121]
	RCTs	Patients with CHF	NA	0.72 (0.63–0.83) vs. placebo		0.80 (0.68–0.93) vs. placebo	[122]
	RCTs	Patients with HF	NA	0.72; *p* < 0.00001 vs. placebo	0.83; *p* = 0.004 vs. placebo	0.86; *p* = 0.03 vs. placebo	[123]
Canagliflozin	Cardiovascular outcome trials	Patients with type 2 diabetes	NA	0.64 (0.53–0.77) vs. placebo	NA	NA	[117]
	RCTs	Patients with type 2 diabetes	NA	0.64 (0.53–0.77)	NA	0.84 (0.72–0.97)	[124]
Ertugliflozin	Cardiovascular outcome trials	Patients with type 2 diabetes	NA	0.70 (0.54–0.90)	NA	NA	[117]

* Hazard ratio (95% confidence interval). ACM, any cause mortality; CHF, chronic heart failure; CVM, cardiovascular mortality; HF, heart failure; HHF, hospitalization for heart failure; NA, not available; RCTs, randomized controlled trials; Ref., reference.

**Table 3:** The risk reduction in renal outcomes owing to SGLT2is in randomized controlled trials using patients with type 2 diabetes and/or CKD.

RCTs	Study Protocol, Used SGLT2i and Patients Studied	Incident or Worsening Nephropathy	Doubling of Serum Creatinine Level	Initiation of Renal- Replacement Therapy	Progression of Albuminuria	Renal- Specific Combined Outcomes	ESRD	Ref.
EMPA-REG OUTCOME	RCT, Empagliflozin, Patients with type 2 diabetes and CVD	−39%	−44%	−55%	NA	NA	NA	[5]
EMPA-REG OUTCOME (Asian Patients)	RCT, Empagliflozin, Patients with type 2 diabetes and CVD	−36%	−36%	NA	NA	−52%	NA	[137]
EMPRISE East Asia study	Databases, propensity score-matched analyses comparing with DPP4i, Empagliflozin, Patients with type 2 diabetes	NA	NA	NA	NA	NA	−63%	[115]
DECLARE- TIMI 58	RCT, Dapagliflozin, Patients with type 2 diabetes	NA	NA	NA	NA	−24%	NA	[4]
DAPA-CKD	RCT, Dapagliflozin, Patients with CKD	NA	NA	NA	NA	−44%	NA	[138]
DAPA-CKD	RCT, Dapagliflozin, Patients with CKD	NA	−32%	NA	NA	NA	NA	[139]
CANVAS Program	RCT, Canagliflozin, Patients with type 2 diabetes	NA	NA	NA	−27%	−40%	NA	[2]
CREDENCE	RCT, Canagliflozin, patients with type 2 diabetes and albuminuric CKD	NA	NA	NA	NA	−34%	−32%	[6]

Renal-specific combined outcomes include a sustained reduction in the estimated glomerular filtration rate, the need for renal-replacement therapy, death from renal causes, end-stage kidney disease, and a doubling of the creatinine level. CKD, chronic kidney disease; CVD, cardiovascular disease; ESRD, end-stage renal disease; NA, not available; RCT, randomized controlled trial; Ref., reference.
